# Unpacking Partnership, Engagement, and Collaboration Research to Inform Implementation Strategies Development: Theoretical Frameworks and Emerging Methodologies

**DOI:** 10.3389/fpubh.2018.00190

**Published:** 2018-07-11

**Authors:** Keng-Yen Huang, Simona C. Kwon, Sabrina Cheng, Dimitra Kamboukos, Donna Shelley, Laurie M. Brotman, Sue A. Kaplan, Ogedegbe Olugbenga, Kimberly Hoagwood

**Affiliations:** ^1^Department of Population Health, New York University School of Medicine, New York, NY, United States; ^2^Child and Adolescent Psychiatry, New York University School of Medicine, New York, NY, United States

**Keywords:** engagement, collaboration, partnership, patient engagement, patient-centered, community engagement, team science, implementation strategies

## Abstract

**Background:** Partnership, engagement, and collaboration (PEC) are critical factors in dissemination and implementation (D&I) research. Despite a growing recognition that incorporating PEC strategies in D&I research is likely to increase the relevance, feasibility, impacts, and of evidence-based interventions or practices (EBIs, EBPs), conceptual frameworks and methodologies to guide the development and testing of PEC strategies in D&I research are lacking. To address this methodological gap, a review was conducted to summarize what we know, what we think we know, and what we need to know about PEC to inform D&I research.

**Methods:** A cross-field scoping review, drawing upon a broad range of PEC related literature in health, was conducted. Publications reviewed focused on factors influencing PEC, and processes, mechanisms and strategies for promoting effective PEC. The review was conducted separately for three forms of partnerships that are commonly used in D&I research: (1) consumer-provider or patient-implementer partnership; (2) delivery system or implementation team partnership; and (3) sustainment/support or interagency/community partnership. A total of 39 studies, of which 21 were review articles, were selected for an in-depth review.

**Results:** Across three forms of partnerships, four domains (cognitive, interpersonal/affective, behavioral, and contextual domains) were consistently identified as factors and strategies for promoting PEC. Depending on the stage (preparation or execution) and purpose of the partnership (regulating performance or managing maintenance), certain PEC strategies are more or less relevant. Recent developments of PEC frameworks, such as Partnership Stage of Change and multiple dynamic processes, provide more comprehensive conceptual explanations for PEC mechanisms, which can better guide PEC strategies selection and integration in D&I research.

**Conclusions:** This review contributes to D&I knowledge by identifying critical domain factors, processes, or mechanisms, and key strategies for PEC, and offers a multi-level PEC framework for future research to build the evidence base. However, more research is needed to test PEC mechanisms.

## Background

### Introduction

Dissemination and Implementation (D&I) research, which involves the use of diverse strategies to facilitate adoption, integration, and sustainability of evidence-based interventions and practices (EBIs/EBPs) in diverse settings, is a rapidly growing field in health research (1). The successful development, implementation, dissemination, and sustainability of EBIs/EBPs requires communication, collaboration, and consensus among all involved, including consumers (end users), implementers, and related partners who contribute to sustainability of EBIs/EBPs. To accomplish this, Partnership, partner Engagement, and Collaboration (PEC) have been identified as critical strategies in D&I research (2, 3). In a recent compilation of recommended strategies by D&I experts, more than one-third were PEC-related strategies (e.g., coalition building, creating a learning collaborative, developing academic partnerships, involving patient/consumers and family members, organizing clinician implementation meetings, and promoting network weaving) (3). Despite the importance of PEC strategies and their potential contribution for improving service implementation and health outcomes at the individual, community, and population levels, the conceptualization and methodologies for studying PEC are not well defined and not well integrated into D&I research. Although PEC research has been applied in multiple fields over the years, including military, business, sports, academia, and health, lessons learned, and findings from these fields have not been systematically applied to inform PEC strategies for enhancing EBIs/EBPs in implementation research.

### Multilevel partnership, engagement, and collaboration in D&I research

In D&I research, PEC can be applied across different programs and interventions (4) and with diverse partners form multiple levels (5). Partners involved in D&I usually include consumers, a team of providers/implementers (i.e., those who provide EBIs/EBPs), and a team of multi-disciplinary partners (i.e., those who set up structures and policies, and provide support for implementation and sustainment of EBIs/EBPs). The purpose of developing strong PEC in D&I research is to build support across the individual, team, and organizational levels to work toward common goals for EBIs/EBPs, use or share skills and resources to implement EBIs/EBPs, and seek input and support of experts from different disciplines. Therefore, D&I research requires consideration of PEC strategies for multiple forms or multiple levels of partnerships.

At the consumer-provider level (consumers also defined as patients or targets of EBIs/EBPs, and providers also defined as implementers who provide EBIs/EBPs), PEC between consumers and providers is critical because substantial research has documented that an effective patient-provider relationship can optimize the patient's use of intervention strategies and engagement in treatment (6, 7). Greater patient-centered care or patient-provider partnerships are associated with better patient outcomes, including increased health knowledge, management skills, competency, self-efficacy, and sense of control and wellbeing over personal health, and well-being (8). Additionally, better patient-provider partnership also benefits providers because of increased patient satisfaction with their care (8). Therefore, application of PEC strategies to promote patient-provider partnerships has implications to improve EBIs/EBPs acceptability and patient-centered care outcomes, and to enhance patients' use of EBI health promotion and management strategies.

At the EBI/EBP delivery system level (or implementation team level), quality of interaction, relationship and behavioral processes of implementation team members can influence teams' performance and effectiveness in EBIs/EBPs implementation (9, 10). Partnership and implementation barriers that are commonly identified at this level include: lack of effective communication and coordination among team members, lack of sufficient buy-in from team members, high turnover, failure of partnership leaders to engage team members, lack of sufficient funds to support partnerships, and team member burnout (11). Therefore, PEC strategies that engage teams' long term collaborative efforts, and empower and motivate members to proactively problem-solve partnership barriers may enhance team efficiency and the quality of EBIs/EBPs implementation (9, 10).

At the sustainment/support system level, D&I research requires consideration of the sustainment and sustainability of EBIs/EBPs. Sustainment is the continued use of EBIs/EBPs within practice settings (12); sustainability is the extent to which the EBIs/EBPs can be delivered with their “intended benefits” over an extended period of time after external support from the donor agency terminates (13). Thus to support sustainment and sustainability, D&I research requires PEC between implementation team members and external partners (e.g., patient advocates, EBI/EBP providers, funders, researchers, institutions, community-based organizations, relevant policymakers, and healthcare system partners) (4, 14). Such cross-disciplinary and cross-organizational partnerships address potential structural and system-level barriers and to the expansion and sustainability of EBIs/EBPs (5). Partnership barriers that commonly occur at this level include conflicts between: (1) priorities and competing demands across organizations or communities; (2) leaders' and partners' roles; and (3) models of partner/ community relationships (15). Therefore, utilizing PEC strategies to address conflicts and promote partner engagement and cross-disciplinary partnerships will have important implications for gaining greater support in implementing and sustaining EBIs/EBPs.

### The study aims

While partnerships in D&I research commonly occur at multiple levels, there has been no multi-level conceptual model to guide PEC strategy development or testing. PEC research is often carried out separately for different partnership levels, and commonly focuses on one level at a time. To inform the development of an integrated D&I framework for PEC strategies, it is important to understand and summarize current research on each partnership level, especially related to the core components and theoretical processes that contribute to effective PEC strategies. Thus, the overall goal of this paper is to address D&I knowledge gaps by reviewing PEC literature and synthesizing knowledge to guide the development of a multi-level PEC theoretical framework. The review focuses specifically on PEC factors influencing PEC processes and outcomes, theoretical frameworks, and evidence from testing of PEC strategies. Given that the central component of the partnership is interpersonal relationship building, we expected that the literature would identify core components that work across different levels of partnerships. The review was therefore synthesized separately for the three levels of partnership. This paper was not intended to be an exhaustive review of the literature, but rather to provide a high-level view of the approaches in which multi-level PEC strategies are studied in D&I contexts.

## Method

### Definitions

Terms related to PEC have been widely used interchangeably and inconsistently. For the purpose of this study, definitions from an array of review papers, as detailed below, were applied to guide our review (5, 14, 16). Review papers were selected based on the inclusion criteria described in the Method section.

#### Partnership

In D'amour et al.'s review paper, partnership is defined as “two or more *actors* join[ed] in a collaborative undertaking (or a set of common goals and specific outcomes) that is characterized by a collegial like relationship that is authentic and constructive.” A partnership can be a relationship between as few as two partners or it can involve a larger number of individuals from groups and organizations (e.g., a network, coalition, or consortium) (5). Under this definition, partnership research has focused on approaches to developing partnerships (e.g., formalizing, sustaining, and ending partnerships) and strategies to build strong working relationships (e.g., cognitive, emotional, and behavioral strategies) (17).

#### Engagement

Based on Concannon et al.'s review, engagement is defined as “A bi-directional relationship between the patient (or consumer, family) and provider (or implementer) or between the partner and researcher that results in informed decision-making about the selection, conduct, and use of research or interventions” (14). Under this definition, engagement research has focused on strategies to build strong bi-directional relationships between the partners that enhance trust, commitment to collaborate, shared decision-making, problem-solving, and behavioral changes. The terms engagement and alliance are often used inter-changeably (14).

#### Collaboration

Based on Mattessich et al.'s review (16), collaboration is defined as a mutually beneficial and well-defined relationship entered into by two or more organizations to achieve common goals. Collaborations include a commitment to mutual objectives, a jointly developed structure, shared responsibility, mutual authority, and accountability for success, and sharing of resources and rewards (16). Under this definition, collaboration in research has focused on strategies to enhance partners' ability to work together to achieve mutual benefits (17). Collaboration is conceptualized as distinctive from cooperation and coordination, which represent earlier stages of organizational partnership (16). Specifically, cooperation is characterized by informal relationships (that exists without any commonly defined mission or planning effort), informal information sharing, preserved authority in each organization, and separated resources by organizations. Coordination is characterized by a more formal relationship, an understanding of compatible missions, with some planning and division of roles, and some established communication channels (16).

Taken together, based on the listed definitions, partnership can be conceptualized as a broader umbrella term that includes engagement and collaboration. Partnerships can occur in multiple forms and at different levels. Therefore, in our review, we included inter-related PEC literatures and diverse types and forms of partnerships.

### Literature review methods

A cross-field scoping review was conducted, and the review was carried out separately for three forms of partnerships that are commonly applied in D&I research (described above). The scoping review method was used because it provides a useful initial approach to generate foundational knowledge (for each level of PEC research), and to inform approaches for future systematic review (18). In the scoping review, the 5-step method outlined by Arksey and O'Malley(18) was applied. The 5 steps include: (1) identifying the research question (i.e., factors and processes for three levels of PEC); (2) identifying relevant studies/literature; (3) study selection; (4) charting the data; and (5) collating, summarizing, and reporting results. The overall inclusion criteria of articles for this review included studies that: (1) examined partnership, engagement, and/or collaboration factors, processes, mechanisms, or effectiveness of strategies; (2) examined diverse types and forms of partnerships; (3) had health implications; and (4) were published in English language, peer reviewed literature, from 2000 to 2017, and in PubMed, Ovid MEDLINE, or by credible federal research institutions (e.g., NIH, AHRQ, CDC). Studies that only characterized or described partnership development approaches, and did not examine factors associated with PEC, discuss theoretical frameworks, or assess partnership outcomes were excluded.

To understand consumer-provider level PEC, the literature about patient-provider partnerships, patient/family-centered care, patient/family engagement research that focuses on factors that influence PEC processes and outcomes, and intervention strategies for promoting consumer-provider relationships and engaging consumers to actively use EBI strategies were reviewed. For delivery-system-level PEC, relevant literature about team collaboration, teamwork, inter-professional collaboration, and teamwork interventions that focused on factors that influence PEC processes and outcomes, and intervention strategies for effective team partnership and teamwork were reviewed (9). To understand sustainment/support system level PEC, the literature about multidisciplinary collaboration, quality improvement collaboration, patient-centered outcome research (PCOR), patient/community participation in research, community-based participatory research (CBPR), and collaborative/team science research that focused on factors that influence PEC processes and outcomes, and intervention strategies for effective collaboration across diverse organizations and disciplines were reviewed. These themes were considered because they included diverse partners from multiple organizations, emphasized equitable partnership building, studied factors related to development and sustainment of collaboration, and considered complexity in collaboration process (14, 19–24).

**Figure 1:**
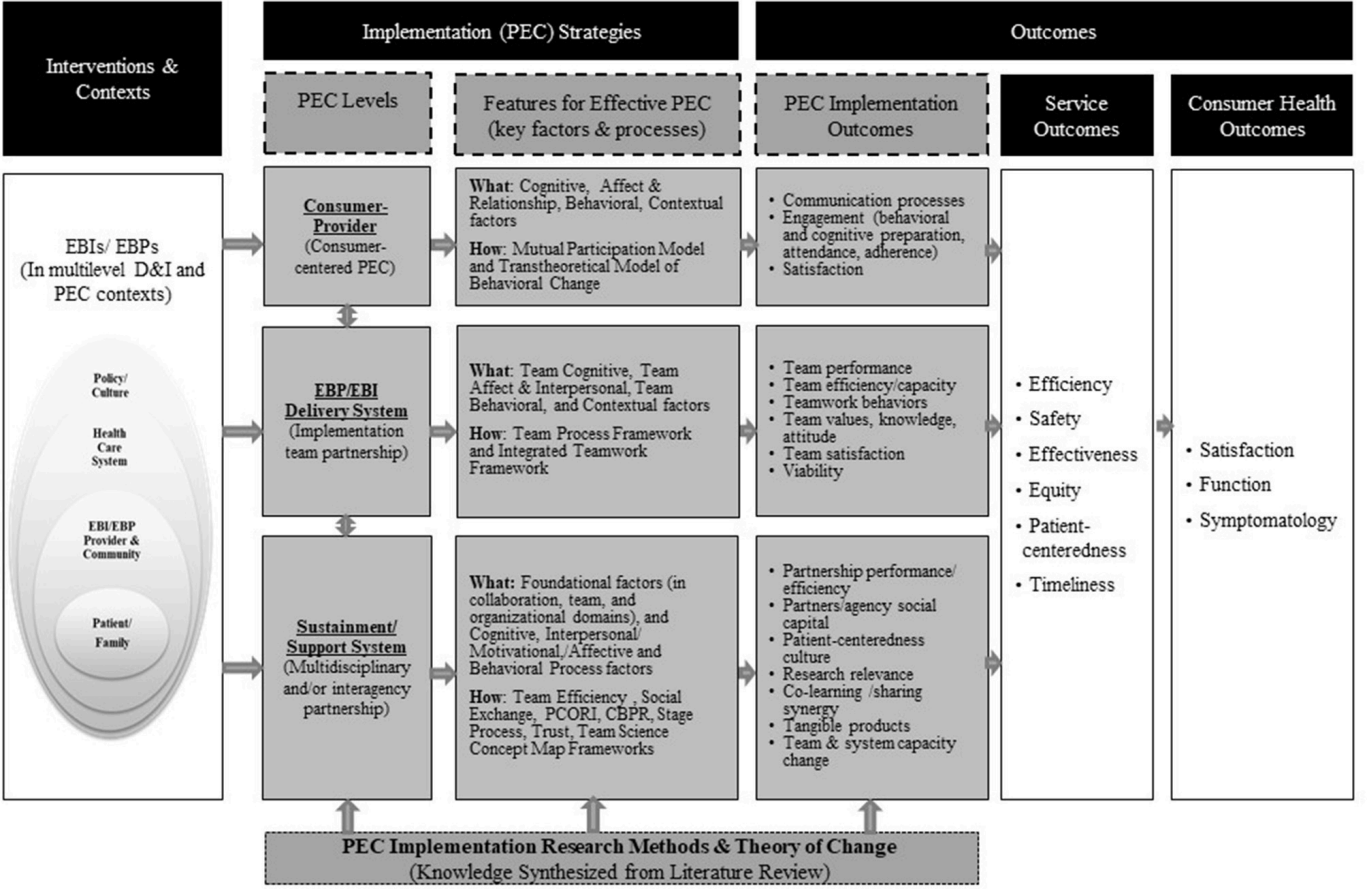
An integrated multilevel partnership, engagement, and collaboration framework for D&I research. Model adapted from Proctor et al.'s (25). Model of implementation research. Each level of PEC represents various types of partnerships. The consumer-provider level includes partnerships between consumers or targets of EBIs/EBPs and implementers who provide EBIs/EBPs. The EBI/EBP delivery system level includes implementation team members from EBIs/EBPs. The sustainment/support system level includes partners for sustainment and sustainability of EBIs/EBPs. Description in boxes “Features for effective PEC” and “PEC implementation outcomes” resulted from the PEC literature review.

Figure 1 shows the multilevel PEC conceptual framework that guided this literature review [adapted from Proctor et al. (25)]. The gray boxes represent a summary of the findings from the content synthesis.

## Results

Tables 1S–3S in the Supplemental file document the charting of review data in detail for studies included in the three levels of PEC literature. Below, findings for each level of partnership are synthesized. Each section is divided into a description of factors that influence PEC processes and outcomes, frameworks used to study PEC strategies, and studies that tested PEC strategies and impact evidence for PEC strategies. A very large body of PEC related studies met our inclusion and exclusion criteria (see number below for each level of PEC review); therefore, priority was placed on review papers, when available. Reviews that examined features for effective PEC and provided approaches for assessing PEC outcomes were included first. Additional selected articles were added when predictors and processes for effective PEC were not covered in the reviewing articles. Figure 1 and Table 1 show a summary of key results from the cross-level PEC literature review, based on the included articles.

**Table 1:** Summary of literature review on PEC related factors, frameworks, and intervention strategies.

	**Consumer-Provider level PEC**	**Implementation Team level PEC**	**Sustainment/Support System Level PEC**
**Frameworks**	**The mutual participation model**: Effective patient-provider partnership is developed through mutual participation and approximately equal power in treatment process **The transtheoretical model of behavioral change (TTM)**: Consumer-provider level PEC and patient health behavior change are guided by a staged approach of change	**Team process framework:** Teamwork is developed through a process of Multilevel Contexts 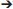 Team Processes (team cognitive, interpersonal, affective, motivational, and behavioral) 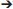 Team Effectiveness **Integrated framework**. Effective team PEC considers two domains of behaviors that function to *regulate a team's performance* and *manage team maintenance* (keeping the team together)	**Team efficiency framework:** Multidiscipline PEC is developed through a process of Contexts 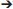 Processes 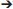 Collaboration Outcomes **Social exchange theory:** An individual/agency joins a group for exchange purposes **PCOR framework:** Effective PEC considers (a) foundation factors (awareness of PCOR method, value for patients); and (b) engagement and action of PEC factors **CBPR framework**. Effective PEC considers contextual factors and group dynamic factors (structural, individual, relational dynamics) **Stage process framework:** Factors for effective PEC depend on the partnership stage **Trust framework**. PEC outcomes hinge largely on trust and 3 related factors (openness, transparency and diversity) **Team science concept map framework**. Effective partnership requires consideration of team factors, support factors, and meta factors
**Features for effective PEC** (Factors influence PEC behaviors, processes, and subsequent PEC outcomes)	•***Multi-Level contextual factors:*** e.g., service environment, resources, individual partner characteristics •***Cognitive factors:*** e.g., recognizing patients' perspectives and experiences, assessing patients' strengths, and needs, providing information using individualized approach •***Affect and interpersonal relationship factors:*** developing trust, caring, empathetic, respectful, supportive relationships; building a partnership alliance; identifying and handling emotional problems •***Behavioral factors:*** sharing control of the decisions and responsibility; providing support; taking action to increase EBI/EBP accessibility, and for finding and trying out solutions to address problems or increase participation; and reinforcement management	•***Multilevel contexts:*** see contextual domain in consumer-provider level PEC •***Team cognitive process factors:*** e.g., team mental model, team learning, commitment •***Team interpersonal, motivation, affective process factors:*** e.g., team cohesion, team efficacy, team affect, team conflict, psychological safety •***Team behavioral process factors:*** (i) factors related to regulating team performance (e.g., goal-specific planning before a team task; task-related collaboration and team monitoring during team task execution; and team practice innovation after team task); *(ii)* factors related to team management (e.g., support and conflict management)	•***Collaboration foundation factors:*** (i) Collaboration environment (e.g., history, interdependence, flexibility, reflection on process, collective ownership); (ii) Team composition (e.g., team diversity, disciplinary dynamic, team membership); and (iii) Organization characteristics (e.g., resources, staff, time, incentives, skilled leadership) •***Factors by processes of collaboration:*** (i) Cognitive process factors; (ii) Interpersonal, motivational and affective process factors; and (iii) Behavioral process factors (similar to factors listed under Implementation team-level PEC factors/strategies) •***Factors by stage of collaboration:*** (i) During the preparation stage may include interpersonal and operational related factors (e.g., sharing goals, setting the stage for an engaged and supportive organizational culture, developing institutional structures to address and support potential system barriers, mutual respect, building partners' knowledge and skills); and (ii) during the execution stage may include partnership synergy, knowledge exchange, monitoring, and ongoing support-related factors (e.g., co-learning strategies, building reciprocal, equal, genuine, and trust-worthy relationships, re-assessment and feedback)
**PEC intervention** (Strategies tested)	**Provider training strategies**: PEC interventions have focused on psychological and relationship aspects of communication style, helping providers gain skills in identifying and handling patients' emotional problems, sharing decision-making power with patients, and utilizing personalized-care that uses empathy and seeing each patient as a whole and unique individual **Patient strategies**: PEC interventions have focused on patient engagement, including strategies to promote patients' cognitive preparation, assessment (e.g., assessing patients' barriers to participate, followed with discussion on solutions with patients), and participation (e.g., promoting service accessibility, attendance, adherence)	**Team member training strategies:** PEC interventions have focused on team regulation (strategies to keeping the team together during teamwork preparation, execution, and reflection) and team maintenance (addressing interpersonal dynamics, such as conflict management and psychological support strategies)	**PEC strategies tested in quality improvement collaboration (QIC)**: During QIC set-up, 7 key PEC factors have been targeted and tested (i.e., pre-work-convened expert panel, pre-work-organization commitment, in-person learning sessions, Plan-Do-Study-Act cycles, multidisciplinary team, team calls, email or web support). *During execution period*, strategies such as monitoring data collection, reviewing data for feedback, and external support with monitoring data synthesis have been applied. At the organization level, leadership involvement and providing QI training for members have also been tested. **PEC strategies tested based on the PCOR engagement framework:** 221 PCORI funded projects have tested PCOR recommended strategies **PEC strategies tested based on the CBPR framework:** PEC strategies such as providing CBPR training (to build partners capacity for partnership research), developing structured communication mechanisms to facilitate opportunities for discussion and partnership skills building, and providing technical assistance on research related design have been tested

### Consumer-provider (or patient-implementer) level partnership

More than 5,000 articles related to patient-provider partnership and patient/family engagement were identified, along with several review papers. To avoid redundancy, this review focused on synthesizing findings from eight relevant consumer-provider level PEC review papers (four focused on PEC interventions, four focused on PEC related factors) and two framework papers. In total, the selected 10 studies represented findings from 425 research articles.

#### Factors that influence PEC processes and outcomes

The literature outlined four domains that influence effective consumer-provider partnerships. These include: (a) cognitive domain (e.g., providing knowledge; listening and recognizing patients' perspectives and experiences; assessing patients' strengths and needs); (b) affective and interpersonal relationship domain (e.g., developing trust, caring, empathetic, respectful, supportive relationship; partnership alliance; identifying and handling emotional problems); (c) behavioral domain (e.g., shared decision-making, providing support, actions to increase EBI/EBP accessibility, actions for finding and trying out solutions to address problems or increase participation, and reinforcement management or homework assignment to increase positive behaviors) (6, 8, 26–32); and (d) contextual factor domain (e.g., health service environment, system, and resources; social determinants; individual partner characteristics). The contextual factors influence not only patient-provider partnership behaviors and processes, but also subsequent PEC outcomes (e.g., cognitive benefit, satisfaction, intervention engagement) (28, 30).

#### PEC frameworks

The Mutual Participation Model of Care and Transtheoretical Model of Behavior Change (TTM) model have been applied in studying patient-provider PEC processes. The Mutual Participation Model proposes that patient-provider mutual participation and approximately equal power in the treatment process will increase patients' sense of self-efficacy, improve self-management of health, and increase active participation in treatment (6). The TTM, also known as the Stages of Change Model, proposes that patients move through five stages of change that represent different levels of readiness to engage in behavior change: pre-contemplation/not ready to change, contemplation/getting ready, preparation/ready, action/making change, and maintenance stages. Based on this model, providers are more likely to successfully engage patients in behavior change if they use communication strategies that match recommendations with patients' level of readiness to change (33). For patients in the early stages of TTM, providers may focus on cognitive and affective strategies to build buy-in, awareness, and trust to prepare for change. For patients in the later stages of TTM, providers may focus on behavioral management, support, and motivation strategies to build strong relationship that support and maintain patient's change (33).

#### PEC strategies testing

Based on findings derived from four review articles, which synthesize results from 100 intervention studies, most intervention research has targeted providers and patients separately. In the interventions that targeted providers, most strategies tested were communication and consultation strategies, particularly focused on psychological and relational aspects of communication. Strategies included helping providers gain skills in identifying and managing patients' emotional states, sharing decision-making, demonstrating empathy, and seeing each patient as a whole and unique individual (34, 35). In the interventions that targeted the patients/consumers, most strategies have focused on promoting patients' cognitive preparation (e.g., psychoeducation that promotes knowledge, realistic expectations, and participation in EBIs), increasing use of assessment (i.e., assessing patients' barriers to participate and then discussing solutions with patients), and increasing participation (e.g., promoting access to services, increasing attendance and/or adherence) (26, 36).

#### Impact evidence for individual level PEC strategy testing

Evidence showed that interventions focused on provider communication/consultation style training (with 52 randomized controlled trial [RCT] studies out of 60 studies included in the review papers) resulted in significant impact on improving consultation processes, providers' communication skills, and patient satisfaction (34, 35). However, the effects of such interventions on patient healthcare behaviors and health outcomes were limited. Only complex interventions directed at both providers and patients that included condition-specific educational materials demonstrated greater health benefits for patients compared to single component targeted interventions (34, 35). For interventions focused on patient engagement (with 40 RCT studies included in the selected reviews), researchers found that assessment, strategies that promoted access to services, and psychoeducation were more likely to improve patients' engagement in EBIs (measured by attendance, adherence, and cognitive preparation) compared to interventions that did not use these strategies (26).

### Delivery system level (implementation team partnership)

To understand implementation team member partnerships, literature related to teamwork was reviewed. More than 4,000 articles were identified on team collaboration, teamwork, inter-professional collaboration, and teamwork intervention research. For this review, seven relevant papers were reviewed, four of which were review papers (three focused on factors influencing team PEC or/and processes, and one focused on interventions for promoting teamwork and team performance). The seven reviewed papers represented findings from 434 research articles.

#### Factors that influence PEC process and outcomes and PEC frameworks

Factors that influence PEC processes and outcomes were based on two representative teamwork frameworks (10, 37, 38). Specifically, the Integrated Framework for effective teamwork, developed by Rousseau et al. based on a review of 29 studies that examined teamwork behaviors and processes, proposes that teamwork behaviors are constructed in a nested hierarchical structure (10). Effective team PEC needs to consider two domains: (1) behaviors that function to regulate a team's performance; and (2) management of team maintenance. With regard to regulating team performance, PEC strategies need to include those that occur (a) before team task performance (e.g., creating action plans, team mission analysis, goal setting, cognitive preparation for team task performance); (b) during the execution of team performance (e.g., coordination, cooperation, and information exchange, task-related collaborative behaviors/strategies, monitoring team performance, reflection); and (c) after task team adjustment period (e.g., intra-team coaching, collaborative problem-solving, and team practice innovation). With regard to management of team maintenance, PEC strategies may include psychological support and integrative conflict management (10).

Different from Rousseau's framework (10). which focuses more on PEC strategies based on the stage of team development, Kozlowski et al. (37, 38) proposed a Team Process Framework that posits that the context in which a team works influences team processes, which in turn influence team effectiveness and performance. In Kozlowski's model, team PEC needs to be conceptualized in a multilevel context (considering individual, organizational system, and environmental influences). Moreover, in order to have effective team-level PEC, partnership factors in three distinct but inter-related team processes need to be considered, including: (a) cognitive team processes (e.g., collective team climate and safety climate, team mental models, team learning factors); (b) team interpersonal, motivation, and affective processes (e.g., team cohesion, team efficacy, team affect/emotion/conflict); and (c) team action and behavioral processes (e.g., team coordination/cooperation/communication, team competencies/functions, team regulation, performance dynamics, adaptation) (37, 38).

Authors of other review and theoretical perspective papers (39, 40) also suggest that promoting teamwork requires similar processes to the frameworks described above [e.g., in a review paper of teamwork monitoring instruments, most have focused on team contexts and behavioral processes based on the two conceptual frameworks described above (40)]. In team contexts, team composition and structure, organizational climate, individual attitudes, beliefs, value, and commitment about teamwork were commonly assessed. In assessing team behaviors, collaborative behaviors, such as communication, goal settings, task analysis, monitoring, adjustment collaboration, problem-solving, decision-making, workload sharing, conflict, and team leadership were commonly assessed (40). Team climate, including climate related to psychological safety, team objectives, team commitment, and support for innovation, has also been proposed for fostering effective team PEC and recommended for carefully monitoring (39). These commonly assessed constructs represent the importance of these factors in team PEC processes.

#### PEC strategies testing

In a meta-analysis based on 72 interventions from diverse fields, researchers reported that most intervention strategies have targeted team member training and most content designs were based on the Integrated Framework (10). Strategies commonly included were related to team regulation strategies (e.g., strategies to keep teams engaged during teamwork preparation, execution, and reflection) and team maintenance strategies (e.g., conflict management and psychological support) (9). Other training models applied holistic/humanities and team co-learning approach. This training approach focused on the patient holistic care concept and provided tools and opportunities to facilitate team members' co-learning and inter-professional team collaboration to provide holistic patient care (41).

#### Impact evidence for team-level PEC strategy testing

Authors of the meta-analysis reported that overall, team training had significant, medium-sized effects in enhancing both teamwork and team performance across a variety of team contexts and training methods (9). In addition, regardless of the targeted domains (e.g., preparation, execution, reflection, interpersonal dynamics) and number of teamwork domains targeted, teamwork training significantly improved team performance. However, in terms of improving teamwork behaviors, significant effects only emerged when two or more domains of teamwork were targeted (9). Trainings using the holistic/humanities and team co-learning approach resulted in significant improvements in team efficiency, team value, shared roles, knowledge, satisfaction, and reactions to working in team across all levels of learners (including non-English-speaking and diverse provider staff) (41).

### Sustainment/support system level PEC

To understand sustainment/support system-level partnerships, literature on interdisciplinary collaboration, quality improvement collaboration, patient/community research partnership research (e.g., PCOR, CBPR, patient/community participation in research), and team/collaborative science was reviewed. More than 7,000 articles were identified. For this review, 22 relevant studies were included, 9 of which were review papers and 13 of which were frameworks or empirical studies that examined PEC factors or/and processes. The reviewed 22 studies represented findings from 597 research articles.

#### Factors that influence PEC process and outcomes

Factors and processes for two key topic areas of literature—interdisciplinary collaboration and patient/community-academic partnership research—were examined separately given the rich and diverse topics within each field of research.

Twelve studies that described interdisciplinary collaboration research were reviewed in detail. These included studies on interdisciplinary, quality improvement collaboration [QIC], and team/collaborative science (six were reviews). Overall, results revealed that factors influencing effective PEC mapped onto two broad domains**:** (a) Factors related to team foundation; and (b) factors related to processes. Factors frequently studied under team foundation were related to collaboration environment (e.g., history, political/social climate, interdependence, flexibility, reflection on process, collective ownership, mutual respect, ability to compromise, trust), team composition (e.g., team diversity, disciplinary dynamic, multiple layer of participation, representation of organization), and organization characteristics (e.g., resource, fund, staff, time, incentive, skilled leadership). Better understanding of team foundation factors and PEC contexts can guide the use of PEC strategies to prepare PEC set-up and increase partners' readiness for PEC. Factors frequently studied under the processes were related to cognitive processes (e.g., clear roles, shared visions/values, concrete attainable goals, cross-disciplinary learning), interpersonal/ motivational/affective processes (e.g., established informal relationships, communication mechanisms, value the contribution of collaborators), and behavioral processes (e.g., having open and honest communication, sharing decision-making, power sharing, acknowledging egalitarian nature of relationships, identifying barriers, and problem-solving) (5, 16, 42–47).

Separate from the interdisciplinary collaboration literature, eight studies on patient/community-academic partnership research, including literatures from PCOR, CBPR, and patient/community research partnership research, were also reviewed (four were reviewed studies). Overall, similar PEC factors were identified as in the interdisciplinary collaboration literature, as well as in the implementation team-level partnership literature (described above). However, there were some differences in two areas of research. Patient/community-academic partnership literature was more likely to discuss factors or strategies based on stages of partnership (rather than domains). Factors or strategies frequently studied during the preparation period were interpersonal and operational process related strategies. These might include sharing goals, establishing an engaged and supportive organizational culture, developing institutional structure to address and support potential system barriers, developing mutual respect, and building partners' capacity for partnering skills (22, 24, 48, 49). Factors or strategies frequently studied during the PEC execution period were partnership synergy, knowledge exchange, monitoring, and support related strategies (e.g., co-learning strategies, building reciprocal/equal relationships, assessment, and feedback) (22, 24, 50).

#### PEC frameworks

There are several conceptual frameworks (49) for explaining the effect of PEC at the level of sustainability and support systems. Because of the complexity of partnership at this level, more PEC frameworks have been developed. Conceptual frameworks, such as the Team Efficiency Framework, Social Exchange Theory, PCOR, CBPR, Stage Process Framework, have been applied in interdisciplinary/interagency PEC research (5, 51–54). The Team Efficiency Framework, which is commonly applied in team member partnership (described above), proposes that multi-disciplinary collaboration is a process/configuration of input (contextual factors) process (cognitive, relationship/affective, and behavioral processes) outcomes (performance, innovation, viability) (55). The Social Exchange Theory proposes that an individual/organization joins a group for exchange purposes. The partnership provides specific benefits to individuals/organizations and that, in return, the individuals/organizations are expected to help the group attain its objectives. From this perspective, the challenges that commonly occur during partnerships are related to concerns about power-sharing in attaining equal benefits (56). Therefore, PEC strategies focused on power, decision-making, and interaction dynamics are commonly proposed. The PCOR Framework, developed by the Patient-Centered Outcomes Research Institute/PCORI, emphasizes trust, honesty, co-learning, transparency, reciprocal relationships, partnership, and respect in collaboration processes. PCOR proposes two broader domains of PEC factors to be considered: (a) contextual factors: including internal factors such as awareness of methods for PCOR, a patient centered culture, and external factors (e.g., ways for patients and researchers to partner, resources and infrastructure, policies and governance); and (b) engagement action of PEC factors: including initiating and maintaining partnership; facilitating cross-communication among partners; capturing and optimizing partners' perspective across phases of research; ensuring meaningful influence on research; providing training for partnering; and sharing and applying learnings (52).

The CBPR Framework is also based on trust, respect, mutual benefit, and equitable and shared decision-making principles similar to the PCOR framework (57, 58), and proposes two overarching domains that are relevant to PEC (53, 54). The contextual domain includes contextual factors that influence partnerships, including: social, economic, cultural, local/national governance, policies, and funding trends, role of institutions, historical context of trust/mistrust, both university and community partners' capacities, readiness, and experience in participatory research, and perceived severity of health issue. The group dynamic domain considers three areas of factors that influence PEC dynamics. These include: (a) structural dynamic (e.g., diversity, complexity, formal agreements, real power/resource sharing, alignment with CBPR principles); (b) individual dynamics (e.g., core values, motivations for participating); and (c) relational dynamics (e.g., safety, trust, flexibility in dialogue, listening and mutual learning, leadership influence, power dynamics, self, and collective reflection, participatory decision-making) (54). It is conceptualized that positive collaboration contexts and group dynamics will result in positive synergistic partnerships, appropriate interventions, and research, and improved systems and community capacity (53, 54).

The Stage Process Engagement Framework (proposed by NIH, CDC and other researchers) suggests that key factors for effective PEC depend on the stage of collaboration (51, 59–61). At the initial stage, PEC may focus on clarifying collaboration goals, promoting knowledge about the collaborators, and better understanding the strengths and weaknesses of the partnering contexts. For engagement to occur, it is necessary to visit communities to establish relationship and build trust, and subsequently work toward developing mutual beneficial goals. For engagement to succeed, sharing responsibility, recognizing and respecting the diversity of partners/communities, and creating transparency are necessary. For partnerships to be sustained, mobilizing community assets and strengths, developing the community capacity, resources, and social capital to facilitate creation of innovative strategies, releasing control of action to the community, and being flexible enough to meet changing needs are also needed (51, 59).

Other frameworks derived from team science literature can also be applied to study multi-disciplinary collaboration process. The Trust Framework proposes that successful PEC outcomes hinge largely on the most basic of human relationship “trust.” The nature of complex collaborative relationships is shaped and formed by three trust related factors: openness, transparency, and diversity. High levels of openness (in team social network) and transparency (related to information and knowledge sharing) will foster diversity in innovation. All three factors are required and need to be balanced for the eventual win-win-win success (62). The Team Science Concept Map proposes not only team related factors, but also support and meta factors that influence the performance of PEC need to be considered. Examples for the team factors may include disciplinary dynamic; structure and context for team; and characteristics and dynamics of teams. Examples for the support factors may include institutional support and professional development and management and organization for team. Examples for the meta factors may include definitions of team collaboration and models, measurement, monitoring, and evaluation (63).

#### PEC strategies testing

Some strategies focused on the sustainment/support systems level were identified from the literature on Quality Improvement Collaboration (QIC), and PCOR and CBPR patient/community research partnerships. In QIC research, several PEC strategies have been tested by researchers during QIC set-up and execution periods. Specifically, during QIC set-up, 7 key PEC strategies were commonly tested. These include pre-work-convened expert panel, pre-work-organizational commitment, in-person learning sessions, Plan-Do-Study-Act/ PDSAs cycles, multidisciplinary team, team calls, email, and/or web support). During the execution period, strategies such as monitoring data collection, reviewing data for feedback, and using external support for monitoring data synthesis and feedback have also been applied. At the organizational level, PEC strategies such as involving leadership and providing QIC training for staff members were applied (19). In PCOR research, PEC strategies developed from the PCOR engagement framework were also studied in PCORI funded projects (52). In CBPR research, training strategies based on CBPR framework have also been studied. The goals of these CBPR trainings are to build partners capacity, develop structured communication mechanisms to facilitate opportunities for discussion, develop partners' partnership skills, and capacity, and provide technical assistance on research related design (48).

#### PEC evidence at the sustainment/support system level

Research in this level of PEC strategy testing is limited and relies more on qualitative and short term data collection. A QIC review study (based on 24 RCTs or quasi-experimental studies) found some positive evidence for PEC strategies. In general, the impact of QIC tends to be greater for providers than for patients. At the provider level, about 47% QIC studies showed positive findings (42% mixed findings and 11% no findings) related to patient-centered cares, such as showing improvement on patient health screening/monitoring, use of data to inform interventions, and/or provider teamwork. At the patient-level, only 23% studies showed positive findings (46% mixed findings, and 31% no findings) related to an increase in patients' participation in care or reduction in health symptoms (19). Regardless, findings were not surprising because most QIC focused on provider related PEC strategies.

For PEC strategy testing based on the PCOR framework, findings from a recent study of 221 PCORI funded projects between 2012 and 2016 (based on self-report data from 235 investigators and 260 partners) provide some supporting evidence for the PCOR approach of collaboration. There were 11–52% investigators and partners endorsing improvements on patient-centeredness of study processes and outcomes (e.g., choices of research topics were driven by patients and related to their needs), and 20–81% investigators and partners endorsing improvement in study design, conduct, or/and efficiency (e.g., increasing the appropriateness of research question selection, design, and outcome measures) (52).

Other PEC strategy testing studies have been based on the CBPR framework and have used provider training and technical support to researchers and community partners to promote effective community-academia collaboration. Positive findings have been documented in several studies, especially related to achieving deliverables (e.g., written pilot study proposal, IRB approved study protocol, carried out pilot studies) (48, 64–66).

## Discussion

The purpose of this paper is to describe a PEC framework and methodological gaps in D&I research by reviewing and summarizing findings from a broad range of PEC literature. A total of 39 articles (including 21 review articles) were selected for this review, representing findings from 1,456 research studies. Through this review, factors and theoretical processes that influence PEC, and strategies that promote effective PEC were identified. Findings guided the development of a multi-level PEC framework, which can be applied to strengthen the evidence-based for PEC research in the field of D&I.

In identifying factors for effective PEC, four domains were consistently identified across three levels of PEC. These included cognitive, interpersonal/affective, behavioral, and context preparation (see Table 1 and Figure 1 for summary) (28, 30, 37, 38). Furthermore, certain strategies were found to be more critical based on the partnership stage. In the earlier stages of PEC, cognitive, affective, and experiential behavioral change strategies were more important in order to build buy-in, awareness, mutual goal development, and trust to prepare for partnership (33, 37, 38, 51, 59). In regulating partnership performance, cognitive and behavioral strategies were more relevant, and in management of partnership maintenance (keeping the partnership going), relationship and affective strategies were more relevant (10). In inter-organizational types of partnerships, structural dynamics, and related strategies (e.g., alignment of collaboration goal with agency mission, resource sharing, leadership representation, and power) need to be carefully considered because of their potential influence on relational dynamics (e.g., integration of agency beliefs to team partnership process) (54).

Related to PEC mechanisms, several useful theoretical frameworks that include factors related to PEC processes were identified. For example, the relationship among context-mechanisms-outcome (or input → process → outcome) has been used to develop causative explanations about PEC processes. This approach allows process modeling wherein the outcome of one context-mechanism-outcome becomes the context for the next chain of implementation steps. Although this framework is useful to guide PEC process research, it may not be as useful for studying PEC mechanisms at the sustainment/support system level due to the complexity of behavioral dynamics within and across agencies during different partnership stages. Recently developed integrated frameworks of change, such as the stage of change (e.g., TTM), partnership stage of change (e.g., cooperation-coordination-collaboration; preparation-execution-adjustment) (33, 37, 38, 51, 59, 60), and multiple dynamic processes (e.g., cognitive, interpersonal relationship, behavioral dynamics) (37, 38, 53, 54) may generate more complex and explanatory theories to guide the design of PEC strategies in D&I research.

Leadership is a factor that is frequently studied in D&I contexts. In this review, we found that leadership's function varies based on stages or levels of partnership. At the delivery system level (or implementation team level), team leadership plays a role during the task execution period (to facilitate activity coordination) (40). At the sustainment-level of PEC, skilled/effective leadership is considered to be a collaboration foundation strategy, which plays a supporting role in the interdisciplinary collaboration process and facilitate initial institutional structure set-up to support PEC (15, 16, 51). In inter-organizational collaborations, leadership also plays an important role given the involvement of different partnering organizations (e.g., in negotiation of collaboration goals), and the importance of facilitating cross-communication among agency staff and external partners (5, 19). Therefore, leadership strategies should be considered when they are relevant to study design.

In PEC strategies testing, four approaches were found to be effective. Training (or psychoeducation) for consumers, providers/ implementers, and partnering members was consistently identified as an important PEC strategy across all level of partnerships. Training provides an opportunity for cognitive preparation and skill building to allow partners to communicate more effectively and to actively participate in partnership activities. Training can also target positive relationship building, partnership behavior engagement, and partnership sustainment (9, 26, 35, 41, 48). However, training alone does not change health behaviors. Training that incorporates multiple strategies and targets all partners is more likely to change behavior (9) and to improve patient health benefits (34, 35).

In addition, strategies that focus on assessment/ monitoring/ reflection (e.g., partner members' ability to assess barriers or collect monitoring data for feedback), *participation* (e.g., power sharing, involvement in decision-making), and relationship building (e.g., communication style, conflict management) are useful and could be included in training initiatives (9, 19, 26, 34, 35, 48).

### Implications for D&I research

Three main lessons for D&I research can be drawn from this review. First, researchers may want to consider gathering data about PEC contexts, associated factors, and processes at multiple levels as part of initial assessments of D&I contexts to enable examination of how PEC contexts and processes from each level of partnership contribute to service use, patient health, and sustainment outcomes [as defined by (25)].

Second, several research questions emerged. Most tests of PEC strategies have only evaluated short-term or intermediate outcomes. Limited evidence is available related to long-term effects. In addition, most sustainment/support PEC strategies have only been evaluated as case studies, qualitatively, or in non-experimental designs. Integrated conceptual frameworks have only recently been developed that could elucidate the complexities of analysis of sustainment strategies. Furthermore, the lack of measurement tools for construct assessment has been an impediment. As measurement tools become more refined and feasible, future studies can take advantage of these advances to study these types of strategies.

Third, PEC has not yet been integrated into D&I training. While partnerships are common, true power sharing is rare. Many D&I experts recognize the importance of including PEC strategies, but do not systematically incorporate them into training and evaluate impacts on PEC effectiveness. It would be useful to include training in specific skills related to PEC. These might include team science, community engagement, communication strategies, conflict management, and interpersonal and intrapersonal intelligence (67, 68).

## Conclusion

As population level health issues continue to require complex healthcare policy solutions, it will become increasingly important to improve partnerships, engagement of different constituents, and new collaborations to craft cost-effective and creative solutions on a broad scale. This will entail at a minimum active involvement of patients, policy-makers, providers, community leaders, and researchers. This paper provides several new directions to address D&I knowledge and methodological gaps related to these partnerships. The review and framework not only provides guidance on how PEC related factors and outcomes can be conceptualized, but also how PEC processes can be integrated into more robust D&I designs. As has been reiterated, more research is needed to elucidate both cross and multi-level partnership mechanisms. In particular, systematic and long-term follow-up research will strengthen understanding of PEC strategies to advance EBI/EBPs implementation-effectiveness, sustainability, and system and population health outcomes.

## Author contributions

K-YH, SK, LB, DS, SAK, OO, and KH contributed conception and design of the study. K-YH, SK, and SC was involved in the acquisition, analysis, and interpretation of data. K-YH wrote the first draft of the manuscript. K-YH, SK, SC, DS, DK, and KH contributed to manuscript writing. All authors contributed to manuscript revision, read, and approved the submitted version.

### Conflict of interest statement

The authors declare that the research was conducted in the absence of any commercial or financial relationships that could be construed as a potential conflict of interest.
