# Supplementary material for: Unpacking Partnership, Engagement, and Collaboration Research to Inform Implementation Strategies Development: Theoretical Frameworks and Emerging Methodologies
Source: Front Public Health. 2018 Jul 11;6:190. doi: 10.3389/fpubh.2018.00190 (PMC6050404; doi:10.3389/fpubh.2018.00190)
Supplement: Supplementary file 1 [file Data_Sheet_1.PDF]

APPENDIX. THE SUPPLEMENTAL FILE

Papers included in the review are listed below. A briefly summary of results for each paper is also provided. Table 1S lists papers and findings for the individual-level of PEC literature review. Table 2S lists papers and findings for the delivery-system-level of PEC literature review. Table 3S lists papers and findings for the sustainment/support system level of PEC literature review.

Table 1S. Summary Results for Patient-Provider PEC Research Articles

Author name and year	Aim and Method	Features for Effective PEC<br><br>(Factors & Process)	PEC Outcome Indicators
1 Lewin et al (2001)(32)	• **PEC intervention (provider training)**<br>• To assess impacts of provider training interventions to improve *patient-centered care* and *patient-provider partnership*<br>• **A review study**: included 17 intervention studies (53% RCTs) that focused on training providers to be more patient-centered in clinical consultations. Training contents were determined from providers' perspective	Interventions focused on training providers to be more patient-centered, especially on ***psychological and relationship building aspects*** because, most patient-provider communication in clinical care focuses on health knowledge (disease and management**)**, but ignore psychological and relationship aspects (e.g., psychosocial talk, rapport, ability to motivate/reassure patients), which also have impacts on making health behavioral change or maintaining health.<br><br>**Provider trainings focus on consultation/communication style and psychological and relationship aspects of communication.**<br>• ***Consultation style***: share control of the decision about interventions and management of the health problems with the patient)<br>• ***Developing empathy***: the patients as a whole person who has individual preference situated with social context<br>• ***Identifying and handling emotional problems***	**<u>Outcome indicators</u>**<br>• **Communication/consultation process** (provider communication skills, eliciting patient's explanatory model, clarification of concerns, level of empathy, patients' perception of providers' attentiveness)<br>• **Healthcare behaviors** (concordance with care plans, attendance, service utilization, patient adoption of lifestyle behavior, use of interventions)<br>• **Health status** (psychological measure, clinical measure, quality of life)<br>• **Care satisfaction.**<br>**<u>Impact Evidence</u>**<br>• Most studies showed consultation/communication training improved consultation *processes* and patient *satisfaction.*<br>• There is limited and mixed evidence on the effects on patient healthcare behaviors or health status.
2 Apollo et al., 2006 (6)	• **Study PEC factors**<br>• To identify factors that are necessary to enable effective patient-provider partnership and enable patients to become more integrally involved in the management of health<br>• A qualitative study, based on a *mutual participation model* of care	**Three factors for promoting positive patient-provider partnership** (in HIV care from patients' perspective) **were identified:**<br>• ***Dynamics of provider-to-patient communication:*** communicating what is most important; non-verbal communication<br>• ***Dynamics of patients-to-provider communication:*** patient readiness for intervention, patients can teach providers/share decision-making<br>• ***Dynamics of collaboration:*** recognition of the patients experience, whole person, and the ways in which providers can support and ease its burden	**<u>Outcome Indicators</u>**<br>• Outcomes can be conceptualized from a **"*mutual participation model* of care"**, which proposes that patient-provider mutual participation and approximately equal power in treatment process will increase patients' sense of self-efficacy, improve self-management of health, and increase active participation in treatment.

3 Dwamena et al., 2012 (33)	• **PEC intervention (provider training)**<br>• To assess impacts of the provider training in improving *patient-centered care* and *patient-provider partnership*<br>• **A review study**. An update of Lewin et al' 2001; included 43 RCTs, with 29 new RCTs; 37% studies from USA and 23% from UK	Interventions focused on training providers to use more patient-centered care, especially on psychological and relationship building aspects (see Lewin et al., 2001 for training contents)<br>Interventions were directed at primary care physicians or nurses practicing in community or hospital outpatient settings. Some studies trained specialists. Patients were predominantly adults with general medical problems.	Outcomes were defined similarly to Lewin et al. (2001)<br>**<u>Impact Evidence</u>**<br>• Interventions were effective in transferring patient-centered skills to providers.<br>• The effects on patient satisfaction, health behaviors and health status were mixed. Complex interventions directed at providers and patients that include condition-specific educational materials have more beneficial effects on health.<br>• A new finding for this updated review (compared to Lewin et al., 2001) was that short-term training (< 10 hours) was as successful as longer training.
4 Cunningham & Rosenbaum, 2013 (8)	• **Study PEC factors**<br>• To understand how Measure of Processes of Care (MPOC) has informed patient/ family-centered service<br>• **A review study**: 107 articles from past 20 years of research were included.	**MPOC is a measure that assesses patient-provider relationship and perceptions of the family-centeredness of services received. *5 factors for effective patient-provider partnership (or family-centered care) were assessed:***<br>• *Respectful and supportive care; Enabling and partnership; Co-ordinated and comprehensive care; Providing general information; Providing specific information*	The MPOC long version (56 items)(63)and short version (20 items)(64) have been used in 11 countries, and translated into 14 languages.<br>**<u>Outcomes</u>**<br>Higher scale scores on 5 subscale domains were associated with better patients' mental health, empowerment with the family and empowerment with the service system, professional services, and client satisfaction
5 Lindsey et al., (2014) (34)	• **PEC Intervention (patient focused)**<br>• To identify factors/ strategies of engagement that have been tested in child mental health service research<br>• **A review study**: 38 articles described 40 engagement intervention studies for 89 study groups [receive vs. not receive engagement strategies) were included in the review	**22 engagement strategies and practice factors were identified to promote youth/family participation in care** (at home, clinic, or community settings). <u>Strategies 1-4</u> were the most commonly used.<br>**1 Assessment (66%** of successful groups contained this practice factor/strategy, which using assessments through a variety of methods to understand patients' strengths/needs and during the process the provider can engage patients through *<u>building rapport and an alliance</u>*)<br>**2 Accessibility promotion/strategies to increase participation (51%)** (any strategy used to make services convenient and accessible to increase participation in EBIs/EBPs)<br>**3 Psychoeducation about service/Providing needed knowledge and information (43%)** (review information about services or the service delivery system [e.g., session frequency/contents, roles] with consumers to increase their likelihood for actively participate in EBIs/EBPs.<br>**4 Homework assignment/hands-on exercise (38%)** (to improve consumers' adherence with the goal of reinforcing /facilitating new knowledge or skills that are consistent with the intervention plan)<br>**5 Other strategies (% of successful study groups include the factor/ strategy):** Assessment of treatment barriers (32%); expectation setting (23%); cultural acknowledgement (23%); appointment reminder (19%); therapist reinforcement [e.g., verbal praise] (17%); behavioral contracting (15%); eliciting change talk (15%); relationship/ rapport building (13%); rehearsal/role plays within session exercise (11%); modeling/demonstration of a desired behavior (9%); peer paring (9%); support network (6%); parent copying(6%); case management (6%); motivational (6%); crisis management (4%); therapist response cost/a penalty (4%); and problem-solving [training in the use of techniques to overcome barriers] (2%)	**<u>Outcome Indicators (Engagement intervention outcomes)</u>**<br>• Attendance (attendance at single point or over treatment course, behavior related to attendance, presence of the agreed-upon activities)<br>• Adherence (reflected the patients/families' active participation in a course of collaboratively determined behaviors [in-session participation, out-of-session practice])<br>• Cognitive preparedness (reflected expectations about roles or outcomes, attitudes toward EBIs, understanding of services)<br>• Satisfaction<br>• Barriers to treatment<br>• Enrollment/reach<br>• Clinical outcomes

6	Becker et al., 2015 (26)	• **PEC Strategies & Outcomes**<br>• Examine practice factors that impact treatment engagement outcomes in children's mental health services (study which practice factors for which outcomes)<br>• **A review study**: 89 engagement interventions from 40 RCTs were included	**22 engagement strategies/practice factors were studied** (same factors listed in Lindsey et al., 2014 article). Using distillation and matching method to understand which practice factors were key to *3 domains of engagement outcomes*<br>• **Strategies to increase *attendance/retention***: assessment (68%), accessibility promotion (64%), psychoeducation about service (54%), and assessment of barriers to treatment (54%) were most useful strategies. Other relatively useful strategies were appointment reminders (32%) and homework assignment (39%)<br>• **Strategies to increase *adherence*:** homework assignment (89%), accessibility promotion (78%) and assessment (44%) were most useful strategies.<br>• **Strategies to increase *cognitive preparation*:** Psychoeducation about service (89%), assessment (67%), and modeling (56%), and expectation setting (44%) were most useful strategies	**Engagement outcomes include**:<br>• **Attendance** (presence of the agreed-upon participants during a therapeutic contract)<br>• **Adherence/task-alliance** (an individual's active demonstration in a course of collaboratively determined behaviors, such as session participation, out-of-session participation)<br>• **Cognitive preparation** (understanding of expectation about roles or treatment outcomes, attitudes toward EBI, attributions of causation, motivation for change, perceptions of personal stress and resources, readiness for treatment).
**7**	Ogden et al., 2017(27)	• **Identify PEC factors**<br>• Conceptual mapping of require factors for effective patient-centered care<br>• A qualitative study. Cross-sector stakeholders' perspectives (including health service providers, patients/ families, and policy stakeholders) participated in the concept mapping.	Through conceptual mapping, 123 statements, 13 clusters, and 3 overarching domains were identified as required factors for effective patient-centered care<br><br>***Domain 1: Humanity and partnership***: *Share responsibility* for personalized health literacy; *Patient provider dynamic for care partnership*; *Collaboration* (willingness to become involved); *Share power and responsibility*; *Recognition of humanity-skills and attributes;* Knowing and valuing the patient (partner); *Relationship building* (partnership alliance)<br><br>***Domain 2: Support System, policy and management:*** *Resources for coordination of care; System review, evaluation and new models; Commitment to supportive structures and process; Factors to facilitate change*<br><br>***Domain 3: Education and training:*** *Professional identity and capability development; Explicit education and learning*	Not focused
8	Hill et al., 2017 (28)	• **Identify PEC factors**<br>• Identify key factors for patient- and family-centered cares from patients/families' perspectives<br>• **A review study**, including 49 articles from 44 studies (65% used qualitative/ mixed methods and 35% used quantitative design).	Similar to the factors reported by the Institute for Patients- and Family-Centered Care (IPFCC; www.ipfcc.org), families of pediatric care also identified similar factors.<br><br>• ***Respect and dignity:*** honor families' perspectives and choices; partners' knowledge, values, beliefs, and culture<br>• ***Expressions of compassion and support from providers***: providers' attitude, communication styles, and behaviors that convey the providers compassion, support, caring, understanding of families' experience, and treating patients/families like human beings<br>• ***Information sharing (clarity, usefulness/appropriateness, type, amount, open/honest/humanely style)***: providers communicate and share complete and unbiased information in ways that are understandable, fitting prefer format, affirming and useful for effectively participating in car<br>• ***Participation in care and decision-making (be transparent in decision-making)***: viewing patients/families as experts, and include them in opportunities that they can receive and exchange information with the care team, and participating in decision-making<br>• ***Environment***: physical/structure and partnership/climate environment influence partners/family experience as conveying respect and dignity.<br>• ***Collaboration***: including patients/families in a broader institution/system-wide program development, implementation, and evaluation collaboration.	Not focused

**9**	Richards et al, 2017 (29)	• **Identify PEC factors**<br>• Identify key factors for family-centered care in pediatric hospital care<br>• **A review study,** including 33 articles (29 qualitative and 4 mixed method studies)	**Five main themes** relate to family-centered care were identified.<br>• ***Sharing information (information sharing from providers)*****:** patients/ families want honest, clear, and complete information to be able to actively participate in decision-making and to cope with uncertainty and fear<br>• ***Hearing parental voices/ power of sharing***: families wanting providers to listen to them, answer their questions, address their concern, and incorporate their knowledge into plans. Listening to families is essential for families to be in partnership with providers and to establish a trusting relationships<br>• ***Making decisions with parents***<br>• ***Individualizing communication***: patients have different preferences for communication, such as level of participation, or type and degree of information that is shared. Considering preference will improve partnership.<br>• ***Negotiating roles:*** reducing differential power relationship between providers and families to improve provider-family collaboration	Not focused
10	Higgins et al., 2017 (30)	• **Identify PEC factors**<br>• Concept Analysis for patient engagement<br>• **A review paper**, including 96 articles.	**Four overarching attributes of patient engagement were defined:** 1) personalization of the approach to care (61/96 articles), 2) access to necessary information and resources (55/96 articles), 3) commitment (cognitive and emotional factors) to pursuing quality care (56/96 articles), and 4) provider-patient therapeutic alliance/nurturing the relationship between actors in the encounter (64/96 articles). 30/96 articles contained all 4 areas.<br>**Attributes for engagement were encompassed into 3 general domains:**<br>• ***Attributes of process*:** describe the steps taken by the patient, provider or institution that increasing patient participation in care<br>• ***Attribute of behavior***: represent cognitive or emotional states that stimulate participation in care<br>• ***Attributes of the environment*:** included characteristics of clinical institutions or patient/provider resources that facilitate greater participation in care<br>**Antecedents/associated factors for engagement:** 34 aspects related to patient's experience, provider role, and characteristics of the healthcare system were identified to be associated with engagement.<br>• *Patient related factors:* the most frequent identified antecedents included an individual experiencing ongoing illness or care, participation or invitation to obtain care (37 articles)<br>• *Provider related factors*, the most frequent identified antecedents included organization of health information or care coordination (7 articles), enrollment/scheduling/visit preparation (8 articles), effort toward communication or meaningful patient interaction (13 articles)<br>• *Healthcare institution factors:* antecedents reflecting an effort towards innovation in technology or procedures (12 articles) or an attempt to satisfy new policy standards (10 articles) were identified	**21 outcomes were used to assess engagement outcomes**<br>• Improved outcomes of care and improved patient satisfaction (33 articles)<br>• experience of care included cognitive benefits such as greater understanding and awareness through learning and communication on the part of the patient (30 articles)<br>• Increased patient safety (5 articles)<br>• Reduced costs (3 articles)<br>• Care coordination (5 articles)<br>• identification of best practices (12 articles)<br>• Job satisfaction on the part of care providers through more meaningful interaction and collaboration with patients and other providers (7 articles)

11	Mauriello et al., 2017 (31)	• **Examine PEC Processes**<br>• Provide a theoretical perspective for patient engagement<br>• Apply the Transtheoretical Model of Behavior Change (TTM), also known as the Stages of Change Model, to facilitate patient engagement	The TTM proposes a 5-stage of behavioral changing process. Engaging patients need to consider their behavioral change stage, and different strategies may apply for early and later stages.<br>• **For pre-contemplation**(not ready) **and contemplation** (getting ready) **stages**, ***engagement strategies may focus on cognitive and affective techniques and experiential processes of change*** (e.g., *consciousness raising* [increase pros/resolve ambivalence, dramatic relief, environmental reevaluation]; *social liberation* [realizing that social norms are changing to support the healthy behavior]; *self-reflection* [on current behavior patterns and self-image, and ideas for change]; *self-reevaluation* [realizing that the behavioral change is an important part of one's identify])<br>• **For preparation** (ready)**, action** (making change)**, and maintenance** (keeping up the change) **stages, *engagement strategies may focus on behavioral management, support, and motivation/commitment strategies of change*** (e.g., *self-liberation* [believing in one's ability to change, problem-solving for barriers, and making commitment to change]; *helping relationships* [using social support to make and sustain changes]; *counter conditioning* [substituting healthy alternative behaviors and thoughts for unhealthy ones]; *increase self-efficacy; reinforcement management* [increasing the intrinsic and extrinsic rewards for healthy behavior], and *stimulus control* [removing reminders or cues to engage in the old behaviors, and using cues to engage in the new healthy behavior	**PEC Outcomes**<br>A patient progressing to the next stage of change is a measure of success. Providers can effectively communicate with patient/partners around their behaviors and match engagement strategies to their level of readiness to change.

Note. Literatures in patient-provider partnership/relationship, patient-centered care, family-centered care, and patient/family engagement related to theory/framework or intervention were included.

Table 2S. Summary Results for Team PEC Research Articles

Author name & year	Aim & Method	Features for Effective PEC<br>(Factors & Process)	PEC Outcomes<br>(PEC proximal and long-term outcomes)
1 Rousseau et al (2006) (10)	• **Examine PEC factors and processes**<br>• Examine the conceptual structure of teamwork behaviors and an integration of frameworks<br>• **A review paper** (29 articles discussed domains of teamwork behaviors and team processes were included)	An **integrated teamwork framework** was generated. Teamwork behaviors can be broadly categorized into two broader areas of behaviors<br>***1 Regulation of team performance:*** this can be further divided into 4 subcategories- i) ***preparation*** *of task accomplishment* (team mission analysis, goal specification, and planning); ii) *task-related* ***collaborative behaviors/execution*** (coordination, cooperation, and information exchange); iii) *work* ***assessment*** *behaviors* (performance monitoring and system monitoring), and *iv)* ***team adjustment*** *behaviors* (backing-up behaviors, intra-team coaching, collaborative problem-solving, and team practice innovation)<br>**2** ***Management of team maintenance*** (psychological support, integrative conflict management)<br>• In this integrated framework, the behavioral domains are nested in a hierarchical conceptual structure.	Not focused
2 Kozlowski 2006 & 2017 (35, 36)	• **Examine PEC factors, processes** for effectiveness of teamwork<br>• **A review paper** (based on social and organizational psychology literature (35)<br>• The authors provided an updated perspective in 2017 (36)	Mechanisms for effectiveness teamwork can be conceptualized as "***Team contexts→ Team processes→ Team effectiveness***". The teamwork needs to be studied in a multilevel contexts (considering organizational system, contextual contingencies, and/or environmental dynamics), and include **3 processes.**<br>• ***Cognitive Team processes:*** *Team climate* (collective climate, safety climate); *Team mental models/team transaction memory; Team learning* (capability to interact and too acquire knowledge and skills from each other)<br>• ***Team interpersonal, Motivational, and Affective processes:*** *Team cohesion* (interpersonal cohesiveness, task cohesiveness); *Team efficacy* (collective belief that the group can be effective) ; *Team affect, mood and emotion*; *Team conflict* (task and relationship conflict)<br>• ***Team Action and Behavioral processes***: *Team Coordination, cooperation, and communication*; *Team competencies/functions* (in information exchange, balancing the task load across members, activity pace, monitoring); *Team regulation, performance dynamics, and adaptation* (members' ability to goal-setting and allocate attention and effort around multiple goals)	**<u>Team PEC Outcomes</u>**<br>• **Team Performance**<br>• **Satisfaction** (in meeting team member needs)<br>• **Long term viability** (the willingness of members to remain in the team)
3 Agreli et al., 2017 (37)<br>**(PEC Processes)**	• **PEC Processes**<br>• Examine the link between team climate and inter-professional collaboration (IPC) from a theoretical perspective	**The domains of *climate for work group innovation* include 4 factors**: ***Participative safety*; *Common objectives*** (the accessibility of team objectives and the values related to them); ***Task orientation or commitment to excellence*** (orientation towards agreed team tasks); ***Support for innovation*** (the expectation that each team member, and the team will strive to introduce new practices or improvements in performance).<br>Team climate is based on the assumption that active social construction of climate is initially developed as part of teams, and then becomes integrated at the organizational level.<br><br>**There are overlap between team climate and IPC in the process that foster collaborative practices**<br>• Interaction and communication between team members<br>• Common objectives around which collective work is organized<br>• Responsibility for performing work to a high standard<br>• Promoting innovation in working practices	**Impacts:** Previous research has indicated that team climate acts as a predictor of team effectiveness, quality in healthcare, innovation, and improvement in quality of care and patient satisfaction

4	Brennan et al., 2013 (38)	• **PEC factors**<br>• Compile available instruments that assess teamwork contexts and outcomes (from quality improvement literature)<br>• **A review paper** (451 articles reviewed, 192 articles for content analysis, and 81 instruments for categorization of contents)	Many measures have been identified to assess team contexts, behaviors, process, and outcomes.<br>• ***Team contexts:*** 45 instruments measured *team composition and structure*, *organizational climate for teamwork*, individual *attitudes, beliefs, value, and commitment about teamwork*<br>• ***Teamwork behaviors, processes, regulation of performance:*** 57 instruments measured collaborative behaviors, such as communication, goal settings, task analysis, monitoring, adjustment collaboration, problem-solving, decision-making, participation, cooperation, workload sharing, coordination, conflict, team leadership<br>• However, evidence supporting measurement properties was limited	**Team outcomes**: 59 measured were identified to assess team effectiveness outcomes:<br>• **Task performance outcomes**: perceptions of the team (shared goals) or its effectiveness<br>• **Attitudinal outcomes**: satisfaction with the team, team viability, team climate (e.g., climate for learning, innovation, safety, trust, empowerment), cohesion<br>• **Behavioral outcomes** (i.e., changes to teamwork capability)
5	Hall et al (39)	• **PEC Intervention**<br>• To test a co-learning teamwork intervention (including 4 self-learning modules)<br>• A nested mixed method design was used; comparing impacts between groups with and without learning modules)	**Teamwork training model include:**<br>• **Holistic care** (learn a model of holistic patient case)<br>• **Inter-professional Teamwork:** Identified an inter-professional approach to holistic care; provide tools to enhance interactive learning<br>• **The humanities in health care:** *Human experience* (explores how humans reflect the meaning, value and creativity of the human spirit); *historical perspective* (explore how an illness been treated overtime, and how each profession/discipline has developed and established its role in caring for persons with a particular condition); *ethnics and law; professionalism* (learner's reflections on teamwork in improving care)<br>• **Creative Summary**: Apply the humanities and teamwork framework and write a summary to express what they learned from the experience	**Intervention Outcomes**<br>All levels of learners (pre-licensure or post-licensure, French- or English-speaking, from medicine, nursing, pharmacy, physiotherapy, occupational therapy, social work, spiritual care, or human sciences programs) who participated in Modules show improvement on changing *team efficiency, team value, physician shared roles, knowledge, satisfaction, and reactions to working in team*.
6	McEwan et al (2017) (9)	• **PEC Intervention**<br>• Examine the effectiveness of teamwork training<br>• **A meta-analysis review paper** (51 articles and 72 interventions from military, aviation, academic settings that used controlled experimental designs were reviewed)	**Teamwork interventions (trainings) mainly targeted *regulation of team performance*** (preparation, execution, reflection) or ***management of team maintenance*** (conflict management, psychological support) (based on Rousseau et al, 2006 conceptual model described above)<br><br>**Teamwork Training may be conducted in 4 ways:** *Providing didactic education to team members in a classroom type setting* (lecturing about importance of providing social support); *A more interaction workshop-style format; Involves simulation training,* and *Incorporating team hands-on experience and reviews*	**Outcomes of the teamwork interventions**<br>• Teamwork interventions were shown to be effective at enhancing both **teamwork** and **team performance** across a variety of team contexts and training methods (medium-sized effects)<br>• Team performance improved significantly as a result of teamwork training regardless of the *number of teamwork domains* that were targeted<br>• Significant effects were shown regardless of which domain (i.e., *preparation, execution, reflection, interpersonal dynamics*) was targeted for both teamwork and team performance.<br>• However, in terms of improving **teamwork behaviors**, significant effects only emerged when two or more domains of teamwork were targeted

Note. Literatures in team collaboration, teamwork, inter-professional collaboration, and teamwork interventions were included.

Table 3S. Summary Results for Inter-disciplinary PEC Research Articles

Author name & year	Aim & Method	Features for Effective PEC (Factors & Process)	PEC Outcome Indicators
		**Literature in interdisciplinary, team/collaborative science, and quality improvement collaboration (QIC)**	
1 Mattessich et al. (2001) (16, 65)	• **PEC factors**<br>• A review of research on factors influencing successful collaboration among multiple organizations (based on 281 studies) (16)<br>• Develop and validate a tool for assessing strengths and weakness of collaboration (65)	**20 factors in 6 domains were identified** (from 40 reviewed studies). Number in bracket illustrates number of studies (out of 40) identify the factor<br>**1)Environmental:** *History of collaboration/cooperation ((11), Favorable political and social climate (6); Collaborative group seen as a legitimate leader in the community(3)*<br>**2)Membership characteristics:** *Mutual respect, understanding and trust (27), Appropriate cross section of members/representativeness (18), mutual beneficial(15), ability to compromise (6)*<br>**3) Process and structure:** *Development of clear roles and policy guidelines (15), multiple layers of participation/ or some representation and involvement from every level with each partner organization (17), Flexibility (10), Feel Ownership/ Members share a stake in both process and outcomes (9); Adaptability (7), and Appropriate pace of development (without overwhelming its capacity) (7)*<br>**4)Communication:** *Open and frequent communication (14), established informal relationships and communication links (10)*<br>**5)Purpose:** *Share vision (15); Concreate, attainable goals (10), Unique purpose (collaboration fulfill at least in part for the goal of individual organization)(4)*<br>**6)Resources**: *Sufficient funds, staff, materials, and time (20); Skilled leadership (who are respected by the collaborative partners) (13)*	A 40-item scale (the Wilder Collaboration Factors Inventory) was developed to assess the 20 factors/constructs identified from the review. The same measures can be used as outcomes to monitor changes. More work is needed for scale testing and validation.
2 Bronstein (2003) (40)	• **PEC factors**<br>• Proposed a conceptual model for interdisciplinary collaboration<br>• **A review study,** including multidisciplinary theoretical literatures from social work literature	**The conceptual model articulates five components of interdisciplinary collaboration**: *Interdependence* (reliance on other professionals to meet goals that cannot be met by practicing in isolation), *newly created professional activities* (practice, programs, and systems change and build on the unique knowledge of each collaborator to improve outcomes), *flexibility* (need to blur roles and share power to meet the needs of members). *collective ownership of goals* (shared responsibility for designing and achieving intended goals) , *and reflection on process* (collaborators discuss and use feedback to improve the effectiveness of the partnership)<br><br>***Contextual factors influence collaboration***: Professional role, structural characteristics, personal characteristics and a history of collaboration	Not focused

3	D'amour et al., 2005(5)	• **PEC factors & process**<br>• To identify common concept, theory, and factors influence collaboration<br>• **A review study**, 27 selected papers were reviewed (17 dealt with definition of collaboration, and 10 with frameworks)	**5 factors about collaboration were identified**:<br>• ***Sharing*** (responsibility, decision-making, value, data, planning)<br>• ***Partnership/Relationship*** (openness, honest communication, mutual trust, respect, aware of and value the contributions and perspectives of the other professionals)<br>• ***Interdependency***<br>• ***Power*** (power is shared; each participant whose respective power is recognized)<br>• ***Process*** (dynamic & interactive processes that require negotiation and compromise in decision-making or shared planning and intervention)<br>**Theoretical Frameworks :**<br>• **Organizational theories** (e.g., team efficiency frameworks, which conceptualize collaboration include *input* (contextual factors)→ *process* (leadership, communication, decision-making, value, formalization of collaboration, conflict resolution) → *outcomes* (performance, innovation, well-being, viability)(51).<br>• **Organizational sociology framework**: considers both structuration model of *inter-professional collaboration* (each organization has its own inter-structuring of rules/goals/vision, human relationships, and governance structures) and *inter-organizational collaboration* (different organizations join together to create a collaboration network, which requires developing collaboration rules/goals, collaboration relationships, and governance structures at the network/inter-organization level). Leadership plays an important role in this type of collaboration given their representation of different partnering organizations (5).<br>• **Social exchange theory** (an Individual join a group/partnership for exchange purposes. The partnership provides specific benefits to individuals/organizations and that, in return, the individuals/organizations must help the group attain its objectives; therefore, a stage model may applied- i) assessment and goal setting; ii) determination of a collaborative fit; iii) identification of resources and reflection; iv) refinement and implementation; v) evaluation of performance and feedback (52).	Not focused
4	Weaver et al., 2008 (41)	• **PEC Framework**<br>• Provide a conceptual model of transdisciplinary scientific collaboration framework<br>• **A review study**. The Framework was developed from literature review	**Consider "Antecedents → Processes ←→Outcomes" (processes influence outcomes and vice versa)**<br>• **Antecedents:** include ***intrapersonal*** (dedicated leader, effective leadership), ***social*** (selection of team members, with needed expertise, committed members, development of trust and rapport), ***physical environmental*** (work environment and physical proximity for effective communication and problem-solving), and ***organization and institutional factors*** (some level of institutional change might be required to support multidiscipline collaboration culture, building mentorship capacity, developing mechanisms to reward collaboration activities and reward team problem-solving)<br>• **Process:** include *behavioral* (team problem-solving behaviors), *affective, interpersonal* (building relationship, clear team role, open/respect/positive communication), and *intellectual factors* (publishing transdisciplinary research, recognize intellectual contribution)	**Outcomes** considered include novel ideas, integrative models, new programs, institutional changes, innovative policies

5	Palinkas et al., (2009) (15)	• **PEC factors**<br>• Provide strategies suggestions for effective collaboration based on social marketing and behavioral theories	**Applying social marketing framework to address collaboration barriers between researchers and stakeholders** (e.g., conflicting priorities/values, role perception, relationship, power sharing) because social marketing strategies are generally integrated with behavioral change models, such as the social exchange theory (people are more willing to take an action when they feel efforts will be compensated), self-efficacy, and the trans-theoretical stage of change.<br><br>• **Social marketing collaboration strategies may include: i)** *an <u>awareness</u> of the values and preferences of the consumers of such countermeasures* (cultural exchange); (ii) *<u>communication of the goals and objectives</u>* of countermeasure developers; iii) *a clear delineation of the <u>role</u>* of each participant in the process of countermeasure development, evaluation, dissemination, and implementation; iv) *identification of the <u>barriers</u>* to participation (as described above) *and incentives to overcome these barriers;* and v) an *acknowledgment of the <u>egalitarian</u> nature of stakeholder relationships* in pursuit of a common goal.<br>• **Strategies for constructing a culture of partnership (a minimum 8 steps):** (1) the establishment of a precedent for effective collaboration at the top (*effective leadership development*); (2) the identification of potential change agents (*utilizing champions*); (3) the creation of a common language with the liaisons acting as translators for the various stakeholder groups (*utilizing knowledge broker*); (4) a workshop attended by key representatives from each of the stakeholder groups on how to delineate and make explicit one's hierarchy of values (*buy-in*) (5) the establishment of ongoing opportunities for the exchange of these value hierarchies *(information exchange and negotiation*); (6) change agents and participants in multiple stakeholder working groups should establish clear parameters for engaging in debate and compromise with respect to the prioritizing of behavioral health risks and the development, testing, validation, and implementation of behavioral health countermeasures (*engaging & maintain change*); (7) involve the use of workshops or retreats to facilitate the identification of areas of potential compromise by each stakeholder group (*monitoring barriers and problem-solving*); (8) involve the establishment of clear guidelines for transitioning countermeasures from research and development to dissemination and implementation (*guideline development & broader implementation*).	Not focused
6	Mellin 2009 & 2010 (42, 43)	• **PEC framework**<br>• Provide a conceptual framework for interdisciplinary collaboration(42)<br>• Validate an interdisciplinary collaboration assessment tool (based on the framework) (43)	**Provide an integrated interdisciplinary collaboration framework** that considers:<br><br>• ***Interdisciplinary partnership contexts (Antecedents):*** include *<u>goals of the collaborative practice</u>*; *<u>team components/functions</u>* **(**include Bronstein's 5 components: *interdependence, newly created professional activities, flexibility, collective ownership, reflection on process (40)*; and *<u>composition</u>* (size, demographic, disciplinary backgrounds, stakeholder representation)<br>• ***Processes:*** *Communication, cooperation, coordination (3C)* (most frequently cited process variables for effective collaboration are *<u>accountability, cross-disciplinary training, mutual respect, partnership synergy</u>***)**<br>• ***Other Contextual Factors*** that also influence interdisciplinary processes and outcomes: *Professional role* (expectations about professional roles and responsibilities)**,** *organizational characteristics* (resources, incentive, time, culture)**,** *Personal characteristics, History of collaboration; Social and policy context*<br><br>**Validate the Index of Inter-professional Team Collaboration for Expanded School Mental Health (IITC-ESMH), a 26-item scale for measuring the team composition/function. EFA yielded a 4-factor scale:** (a) *Reflection on Process* (b)	**<u>Outcome Indicators</u>**<br>• Partnership performance (creativity, new solutions)<br>• Partnership (increased collective efficacy, service implementation outcomes)<br>• Organizational/community social capital<br>• Organizational outcomes (e.g., decrease service fragmentation and increase resources, decrease financial burden, additional resources, improved climate)<br>• Satisfaction with services<br>• Improve targeted consumers' health outcomes

			Professional Flexibility, (c) *Newly Created Professional Activities*, and (d) *Role Interdependence*. Cronbach's alphas for the four factors range ,80-.91	
7	Falk-Krzesinski, et al., (2010) (57)	• **PEC Framework**<br>• Proposed a framework for science of team science (SciTS)	**Factors associated team/partnership effectiveness (all of these are study subjects in team science**<br>• **The team factors:** *disciplinary dynamic; structure & context for team; and characteristics & dynamics of teams*<br>• **The support factor:** *Institutional support & professional development for teams; Management & organization for team*<br>• **The Meta factors:** *Definitions of team collaboration & models ; Measurement, monitor & evaluation*	
8	NIH, CDC, CTSA et al (2011)(47)	• **PEC factors and processes**<br>• Summarize community engagement and collaboration principles and effective approaches for collaboration	**Principles for community engagement and collaboration:**<br>***Before starting***<br>1. **Clear about the purpose/goals** and populations/communities you want to engage<br>2. **Become knowledgeable about the community's** culture, social capital, political and power structures, norms and values, demographic trends, history, and experience with efforts by outside groups to engage in various program. Learn more about the community's perception of those initiating the engagement activities.<br>**For engagement to occur, it is necessary to…**<br>3. Go to the community, **establish relationship, build trust**, work with the formal and informal leadership, and seek commitment from community organizations and leaders to create processes for mobilizing the community<br>4. Remember and accept that **collective self-determination** is the responsibility and right of all people in a community. No external entity should assume it can bestow on a community **the power to act in its own self-interest**.<br>***For engagement to success….***<br>5. **Partnering with the community** is necessary to create change and improve health<br>6. All aspects of community engagement must **recognize and respect the diversity of the community**. Awareness of the various cultures of a community and other factors affecting diversity must be paramount in planning, designing, and implementing approaches to engaging a community<br>7. Community engagement can only be sustained by identifying and mobilizing community assets and strengths and by developing the community's capacity and resources to make decisions and take action<br>8. release control of actions or interventions to the community and be flexible enough to meet its changing needs<br>**9.** Community collaboration requires **long-term commitment** by the engaging organization and its partners.	
9	De-Graft Aikins et al (2012) (54)	• **PEC factors**<br>• Applying a multidiscipline research partnership framework and test usability of the framework in monitoring partnership in African context	Applying Maselli's et al (2005) (66)*research partnership framework* (that include **11 key factors for success partnership**) to monitor partnership development (in health research).<br>• **11 Factors for effective partnership**: *1) Decide on objectives together; 2) Build up mutual trust; 3) Share information, develop networks; 4) Share responsibility; 5) Create transparency; 6) Monitor and evaluate collaboration; 7) Disseminate the results; 8) Apply the results; 9) Share profits equitably; 10) Increase research capacity;11) Build on achievements*	**Partnership outcome measures include**<br>• Level of engagement: level 1 to 3: involved committing to at least one to 3 goals over the funded life of the project (defined by the team)

		• Understand key ingredients to sustain partnerships	• **Consider collaboration continuum (stages):** starting partnership (start as grants) → transactional stage (combine resource toward a common goal) → Integrative stage (partnership resources are merged into a new identify)<br>• **Key ingredients for sustain partnership include:** S***ocial capital*** (share understandings, values, and links individuals and groups share that engender trust and collaboration; ***Measurable goals*** (clear, realistic, and measurable); ***Administrative support*** (non-technical aspects of partnership activities); ***Creative and innovative strategies*** (openness to new ways of using existing resources and to securing additional resources); and ***Funding***	
10	Nadeem et al (2013) (19)	• **PEC Interventions**<br>• To identify factors/ strategies that have been tested in health service quality improvement collaborative research<br>• **A review study** (for selected 24 QIC intervention, using RCT or quasi-experimental designs)	• **7 key Quality improvement collaborative (QIC) <u>components/strategies</u>** were tested (% indicates proportion of studies/out of 20 reviewed studies applied the component in QIC set-up): *Pre-work-convened expert panel (25%); Pre-work-organizations required to demonstrate commitment (15%); In-person learning sessions (100%;* didactic training in a particular care process or practice; training on PDSAs, foster team planning and sharing experience*); Plan-Do-Study-Act (PDSA) cycles (75%); Multidisciplinary QI team (70%); QI team calls (70%); Email or web support (60%)*<br>• **3 factors of QIC <u>processes</u>** were identified (describes how PDSAs and other QI activities were conducted): *Sites collected new data for QI (75%*; e.g. performance indicators, target outcomes, feedback data*); Sites review data and used feedback (40%); External support with data synthesis and feedback (45%)*<br>• **Organizational involvement (**pertains to indicators of how the QIC penetrated different levels of the organization)**:** *Leadership involvement/outreach (45%); Training for non-QI team staff members by experts (10%) or by QI team(30%)*	On average, 6-7 components/strategies were included in intervention<br><br>**<u>Impacts of QICs</u>**<br><br>Most outcomes were derived from medical records or administrative data that did not directly assess change in provider behaviors.<br><br>Studied have found support in relation between QIC components and study outcomes<br>• <u>At the provider level:</u> 47% studies show positive findings, 42% showed mixed findings, and 11% show no findings<br>• <u>At the patient-level:</u> 23% studies show positive findings, 46% showed mixed findings, and 31% show no findings<br>• The greatest impact of QICs was at the <u>provider-level</u>. Patient-level findings were less robust
11	Adams et al. (2014) (56)	• **PEC framework**<br>• Proposed a framework for collaborative science	**The successful outcomes hinge largely on the most basic of human relationship- "Trust".** The nature of complex collaborative relationships will be shaped and formed by *three factors—**openness** (within team social networks), enables **transparency** (on knowledge sharing), which fosters **diversity** (in innovation).* All three factors are required and will need to be balanced for the eventual win-win-win success for developing trusted collaborating teams.	

12	Cooke et al, 2015 (44)	• **PEC factors & processes**<br>• Understand how do individual factors (openness to divergent ideas) influence team process (e.g., cohesion)<br>• Understand how do both individual and team dynamic influence the effectiveness and productivity of team<br>• **A review** on Team Science	<u>**Factor associated interdisciplinary teams' Effectiveness**</u><br>• ***Cognitive team process: T****eam mental models/ team transaction memory* (share understanding about task requirement, procedures, and role responsibilities)*; Team learning/cognitive team interaction* (decision-making, problem-solving, situation assessment, planning, knowledge sharing); *Team climate* (climate that related to learning)*;Psychological safety*<br>• ***Motivational and Affective Team Process:*** *Team cohesion; Team efficacy; Team conflict*<br>• ***Team Behavioral Process:*** *Team process competencies* (e.g., interpersonal knowledge/ management knowledge; preparation/task engagement & reflection/ conflict management/motivation)*; Team self-regulation*<br>• **Team composition factors:** ***Team diversity*** (racial, gender, country diversity); ***Group faultlines*** (hypothetical divisions within a team; such as task-relevant, expertise, and demographic divisions); ***Subgroup in teams*** (subset of team members who are uniquely interdependent in some way; e.g., friendship group); ***Changing team membership***	<u>**Outcome indicators**</u><br>• **Cognitive team functioning**: Team identify; Team climate and atmosphere scales; Team skills (team cognitive interaction);<br>• **Motivational and Affective Team Function**<br>• **Team behavioral Function**: Team functioning (cohesion)<br>• **Team Effectiveness**: Team performance (productive, meet requirement)
**Literatures in patient/community research partnership**				
13	Shippee et al., 2013 (46)	• **PEC factors & processes**<br>• To derive a framework for patient and service user engagement (PSUE) in research<br>• **A review study** (based on 202 studies; 41 of these presented engagement processes	<u>**Factors/components of patient and public involvement in research**</u><br>• ***Patient and service user initiation*** (include stakeholders for whom the outcomes are of interest; engage as early as possible; allow partners having active role)<br>• ***Building reciprocal relationships*** (equal partnership, clear roles & respect others' roles; mutual understanding of partners' needs, capacities, and goals)<br>• ***Co-learning process*** (education/training about content/methodology to carry out a productive dialogue or research; provide opportunities to all team members to acquire new knowledge and skills)<br>• ***Re-assessment and feedback*** (evaluating process, and use data to future clarifying roles, expectation, and project modification)<br><u>**Stages/phases of engagement**</u><br>• **Preparation phase** (agenda setting & funding, steering committee; ensuring research is relevant, protocol preparation)<br>• **Execution phase** (study design/procedure/review content, study recruitment, data collection, data analysis/interpretation of findings)<br>• **Translational phase** (finding dissemination, implementation/clinical practice guideline, evaluation of process, plan for future)	Not focused
14	Rodgers et al., (2014) (45)	• **PEC Intervention**<br>• Proposing <u>Capacity building strategies</u> for promote academic-community partnerships	**PEC strategies were tested by focused on capacity building to promote effective collaboration (7 domains were integrated into training)**<br>• ***Share goals*** (team defined share goals and activities that fit organizational cultures)<br>• ***Setting organizational culture that encourage and support community/ stakeholder engaged research***<br>• ***Develop institutional structure and address system barriers to support partnership***<br>• ***Mutual respect*** (established rapport or sense of trust)<br>• ***Provide Human and fiscal resources*** (having staff, monies, and space to carry out the activities)<br>• ***Build partners' Research Knowledge/ or skills*** (a set of skills required to carry out research, D&I implementation)<br>• ***Building parents' capacity for partnering skills*** (a set of skills required to effectively work with other, such as communication, dependability, and transparency)	<u>**Impact Evidence**</u><br>• Using a non-experimental design<br>• Increased collaborative research capacity (by providing training and technical support) has resulted in better achievement in deliverables (e.g., written pilot study proposal, IRB approved study protocol, carry out pilot studies)

15	Jagosh et al., (2015) (67)	• **PEC factors & processes**<br>• To understanding what supports partnership synergy in successful long-term CBPR partnerships; and develop a conceptual model<br>• A qualitative study	• **CBPR focuses on:** ***genuine partnership*** (commitment to co-learning among partners) ***capacity building*** (e.g., involving community or partnering stakeholders in research process); ***knowledge sharing*** (knowledge obtained must be beneficial for all partners), and ***long-term commitment*** **(53, 62)**<br>• Qualitative study support the central importance of CBPR is to **develop and strengthen partnership synergy through trust.**<br>• Sense of trust amongst CBPR members was a prominent mechanism leading to partnership sustainability. Also, trust relationship build overtime (from getting acquainted → experiencing resolving conflict → to trusted partnership)	**Outcomes**<br>• **Highly trusted partnership:** a *high level of commitment* to working out differences; *skills in resolving disputed* and conflict; and *continued maintenance of trust* over time<br>• **Population level outcomes:** (a) sustaining collaborative efforts toward health improvement; (b) generating spin-off projects; and (c) achieving systemic transformations.
16	Belone et al. (2016) (50)	• **PEC framework**<br>• Developed a conceptual/logic model for community-based-participatory-research (CBPR) partnerships<br>• **Review**. Through academic literature review and expert consensus-building to develop the model.	**The CBPR includes 4 overarching domains** (adapted from Wallerstein et al(49)<br>**Contexts:** contextual factors that influence partnerships include *social economic cultural factors*; *local/national governance, policies and funding trends*; *role of institution* (in education, research); *historical context of trust/mistrust*; both university and community *capacities, readiness, & experience in* participatory research; and *perceived severity of health issue*<br>• **Group dynamics:** include ***structural dynamics*** (diversity, complexity, formal agreements, real power/resource sharing, alignment with CBPR principles, & length of time in partnership); ***individual dynamics*** (core values, motivations for participating, personal relationships, cultural humility/identities, personal beliefs/spirituality, community reputation of research team/PI); and ***relational dynamics*** (safety, trust, flexibility in dialogue, listening & mutual learning, leadership influence, power dynamics/stewardship, self & collective reflection, participatory decision-making & negotiation, integration of local beliefs to group process, task roles & communication)<br>• **Intervention & research design change):** see outcome in right column<br>• **Outcomes**: see distal outcome in right column<br>(see Belone et al paper for 2013 revised conceptual model)	**Intermediate Outcomes**<br>• **Intervention & research (design change):** CBPR will improve research and intervention in that i) intervention and research design better *fits local/cultural knowledge, norms & practices*; ii) better *co-learning/ partnership/shared synergy* (i.e., bidirectional translation, implementation, and dissemination); iii) more appropriate *research design* (i.e., research & evaluation design reflects partnership input; more used of community language, instead of expert language)<br>**Distal Outcomes:**<br>• **System & capacity changes:** improvement on policies/practices, sustained cultural-centered interventions (broader reach), power relations/ empowerment/community voices heard, cultural renewal/revitalization, partner/agency capacities (collective reflection & critical thinking)<br>• **Improved Health (distal outcomes)**: decrease health disparities, and improve social justice

17	Drahota et al (2016) (22)	• **PEC factors & processes**<br>• Identify interpersonal and operational factors that influence community-academic research collaborative process<br>• **A review study**; including 50 studies from community-academic partnership (CAP) research (most were case studies using **qualitative methods)**	Based on a-stage model of research-community partnership (68), which **consider 3 phases of partnership: *Formation*** (collaborative process) → ***Execution of activities*** (proximal/process outcomes) → ***Sustainment*** (distal outcomes)<br><br>• **During the formation phase,** collaborative processes need to focus on *interpersonal processes* and *operational processes* and related contributing factors<br>• **During execution phase,** function of partnership should focus on *partnership synergy*, *knowledge exchange*, and *tangible products* **(**proximal outcome)<br>• **During sustainment phase,** collaboration may focus on *improved EBPs/EBIs implementation*, *developed/enhanced capacity to implement EBPs/EBIs*, *improved community care* (policy change, clinical outcome), and *sustainable partnership infrastructure (distal outcomes).*<br><br>**From literature review, 23 facilitating and hindering factors influencing the CAP interpersonal and operational collaboration processes emerged:** Most important factors are *trust; respect among partners; and time commitment)*<br><br>• **12 Facilitators:** 1) Trust (30% studies reported); 2) Respect (30%), 3) Shared vision, goals, and/or mission (26%); 4) good relationship (24%); 5) effective and or/frequent communication (24%); 6) well-structured meetings (19%); 7) clearly differentiated roles/functions of partners (15%); 8) good quality of leadership (11%); 9) effective conflict resolution (9%); 10) good selection of partners (6%); 11) perceive positive community impacts (6%); 12) mutual benefit for all partners (4%)<br>• **11 Hinders:** 1) Excessive time commitment (22%); 2) Excessive funding pressures (17%); 3**)** unclear roles and/or functions of partners (15%); 4) poor communication (13%); 5) inconsistent partner participation (11%); 6) high burden of activities (9%); 7) lack of shared vision, goals (9%); 8) different expectation (7%); 9) mistrust (7%); 10) lack of common language (7%); and 11) bad relationship (4%)	**Outcomes indicators**<br>• **Most focused on proximal outcomes (78%),** such as *partnership synergy (19%), knowledge exchange (26%), or tangible products (72%)*<br>• **Some include distal outcome (33%)** such as *development of or an enhanced capacity to implement programs or intervention (13%), improved community care (19%), sustainable CAP infrastructure (6%), and changed community context (2%)*
18	Duffett 2017 (24)	• **PEC factors & processes**<br>• Examine current knowledge regarding methods and impacts of Patient engagement in research<br>• **A review study** (research synthesis)	**Factors for effective patient engagement in research (patient-academic partnership research)**<br><br>• ***Selection of patient partners***<br>• ***Clearly defined partnership plan*** (a written plan to help team members understand their role, cost and time commitment)<br>• ***Training and ongoing support*** (training for specific research team and patients to allow them to participate and understand discussions**)**<br>• ***Mutual respect and valuing patients' experiential based knowledge and expertise***<br>• ***Start early and continue throughout*** (to have a sense of true ownership)<br>• ***Include a plan of evaluation***	**Impact evidence**<br>• Improved relevance of research to patient priorities, significant contributions to trial design, improved patient information material and/or informed consent documents, improved clinical trial enrollment and decreased attrition, **improved dissemination and/or implementation of research findings**, and increased public trust in research.
19	Goodman et al. (2017) (55)	• **PEC factors & stages**<br>• Identify factors for stakeholder engagement research	**Engagement falls primarily within three broad levels/categories & each categories containing subcategories on the engagement continuum:**<br><br>• ***Non-participation****: Outreach → education*<br>• ***Symbolic participation****: Coordination →Cooperation (limited power in decision-making)*<br>• ***Engaged participation (True engaged participation)****: Collaboration → Patient-Centered → CBPR*	

20	Forsythe et al, 2017 (48)	• **PEC factors & impact evidence**<br>• Present engagement framework used in Patient-Centered Outcomes Research Institute (PCORI)<br>• Evaluate impact evidence from PCORI's funded partner-engagement projects since 2012.<br>• Experiences were reported by 235 investigators (from 221 PCORI projects) and 260 partners/patient (from 124 projects)	**PCORI framework for patient engagement in research**<br><br>• ***PCOR Principles in partnership***: develop trust, honesty, co-learning, transparency, reciprocal relationships, partnership, and respect<br>• ***Foundational factors***: *Internal* (awareness of method for PCOR, value of the patient, perspective interest in PCOR); and *Externa*l (ways for patients and researchers to partner, resources and infrastructure, policies and governance)<br>• ***Engagement (partnership) actions***: *Initiation and maintain partnership*; *Facilitate cross-communication* among stakeholders; *Capture, use and optimize patient/partner perspective* across phases of research; ensure *meaningful influence* on research; *train* for partnering; *Share* and use learnings	**Outcomes (defined in the PCORI)**<br><br>• ***Near-term:*** culture of patient-centeredness in research; meaningful & effective partnership<br>• ***Intermediate:*** relevant research for patients/stakeholders; use of research results in health decisions; quality health decisions; satisfaction with health care experiences<br>• ***Long term*****:** Optimal health<br><br>**Evidence from PCORI projects**<br><br>• ***Enhanced patient-centeredness of study process and outcomes*** (11-52% studies endorse improvement; e.g., research topics are driven by patients and more relevant to the needs)<br>• ***Enhanced study design, conduct, or efficiency*** (20-81% endorse improvement; research questions, design and outcome measures were refined and increase the appropriateness

Note. Literatures in multidisciplinary collaboration, quality improvement collaboration, patient-centered outcome research (PCOR), patient engagement/participatory in research, community engagement/participatory in research, and collaborative/team science research were reviewed.
